# Multiple Layered Control of the Conjugation Process of the *Bacillus subtilis* Plasmid pLS20

**DOI:** 10.3389/fmolb.2021.648468

**Published:** 2021-03-18

**Authors:** Wilfried J. J. Meijer, D. Roeland Boer, Saúl Ares, Carlos Alfonso, Fernando Rojo, Juan R. Luque-Ortega, Ling Juan Wu

**Affiliations:** ^1^ Laboratory 402, Centro de Biología Molecular “Severo Ochoa” (CSIC-UAM), Universidad Autónoma, Canto Blanco, Madrid, Spain; ^2^ ALBA Synchrotron Light Source, Barcelona, Spain; ^3^ Laboratory 35, C. Grupo Interdisciplinar de Sistemas Complejos and Departamento de Biología de Sistemas, Centro Nacional de Biotecnología, CSIC, Madrid, Spain; ^4^ Laboratory B08, Systems Biochemistry of Bacterial Division Lab, Centro de Investigaciones Biológicas Margarita Salas (CSIC), Madrid, Spain; ^5^ Laboratory 216, Department of Microbial Biotechnology, Centro Nacional de Biotecnología, CSIC, Madrid, Spain; ^6^ Laboratory S07, Molecular Interactions Facility, Centro de Investigaciones Biológicas Margarita Salas (CSIC), Madrid, Spain; ^7^ Centre for Bacterial Cell Biology, Biosciences Institute, Newcastle University, Newcastle Upon Tyne, United Kingdom

**Keywords:** quorum sensing, *Bacillus subtilis*, Gram-positive bacteria, antibiotic resistance, horizontal gene transfer, DNA looping, transcriptional regulation, conjugation

## Abstract

Bacterial conjugation is the main horizontal gene transfer route responsible for the spread of antibiotic resistance, virulence and toxin genes. During conjugation, DNA is transferred from a donor to a recipient cell via a sophisticated channel connecting the two cells. Conjugation not only affects many different aspects of the plasmid and the host, ranging from the properties of the membrane and the cell surface of the donor, to other developmental processes such as competence, it probably also poses a burden on the donor cell due to the expression of the large number of genes involved in the conjugation process. Therefore, expression of the conjugation genes must be strictly controlled. Over the past decade, the regulation of the conjugation genes present on the conjugative *Bacillus subtilis* plasmid pLS20 has been studied using a variety of methods including genetic, biochemical, biophysical and structural approaches. This review focuses on the interplay between Rco_pLS20_, Rap_pLS20_ and Phr*_pLS20_, the proteins that control the activity of the main conjugation promoter P_*c*_ located upstream of the conjugation operon. Proper expression of the conjugation genes requires the following two fundamental elements. First, conjugation is repressed by default and an intercellular quorum-signaling system is used to sense conditions favorable for conjugation. Second, different layers of regulation act together to repress the P_*c*_ promoter in a strict manner but allowing rapid activation. During conjugation, ssDNA is exported from the cell by a membrane-embedded DNA translocation machine. Another membrane-embedded DNA translocation machine imports ssDNA in competent cells. Evidences are reviewed indicating that conjugation and competence are probably mutually exclusive processes. Some of the questions that remain unanswered are discussed.

## Introduction

Horizontal Gene Transfer (HGT) refers to the process by which a DNA element/region is transferred from one cell to another. HGT occurs at large scale in bacteria, and a single HGT event can result in the acquisition of several to many genes by a cell and therefore plays a major role in bacterial evolution (Soucy et al., 2015). Unfortunately, HGT also contributes importantly to the dissemination of antibiotic resistance, virulence and toxin genes. The spread of these genes has now become a major global problem, with serious consequences on both human lives and economy (Partridge et al., 2018; CDC, 2019; Lerminiaux and Cameron, 2019). A better understanding of the HGT processes is needed to design drugs or strategies to impede the spread of antibiotic resistance and other pernicious genes. There are three main mechanisms responsible for HGT: transformation, transduction and conjugation (Ochman et al., 2000; Frost et al., 2005; Thomas and Nielsen, 2005; Novick et al., 2010). Of these, conjugation is the mechanism that is majorly responsible for the spread of antibiotic resistance, toxin and virulence genes (Mazel and Davies, 1999; Davies and Davies, 2010).

During conjugation, a DNA element is transferred from a donor cell to a recipient cell; hence, it requires direct contact between the two cells. Conjugative elements are often present on plasmids, but they can also be integrated in a bacterial chromosome. These latter elements are named integrative and conjugative elements (ICE). Conjugation occurs in both Gram-positive (G+) and Gram-negative (G−) bacteria, and although there are important differences (see below), the basics of the conjugation process are conserved in G+ and G− bacteria. Conjugation can be divided into four discernable stages, which have been described in detail previously (Chen et al., 2005; Arutyunov and Frost, 2013; Cabezon et al., 2014; Goessweiner-Mohr et al., 2014; Koraimann, 2018; Li et al., 2019; Waksman, 2019). Below we briefly summarize each stage, to illustrate the large number of proteins involved in conjugation, and the complexity of the process in which the many proteins act together in a temporally orchestrated manner. During stage 1) transfer competent donor cells select and attach to a suitable recipient cell (also known as mating pair formation). This process involves surface-located proteins, some of which are adhesion proteins that establish contact with the recipient cell. The adhesion proteins encoded by conjugative elements present in G− bacteria are generally located at the tip of a pilus structure. Conjugative elements of G+ bacteria do not form so-called sex-pili but probably encode adhesions to fulfill this function. While some proteins are involved in establishing contact, other surface-located proteins serve to inhibit conjugation between two donor cells, in a process named exclusion. In stage 2) the conjugation element is processed to generate the DNA that is transferred to the recipient cell, which in most cases is single-stranded DNA (ssDNA). The DNA processing reaction requires a relaxase, which forms a nucleoprotein complex called the relaxosome that introduces a site- and strand-specific nick within the origin of transfer (*oriT*). Host and/or plasmid-encoded proteins, named auxiliary proteins, often assist in the DNA processing reaction. During stage 3) a sophisticated membrane-embedded translocation machinery, also called transferosome, is synthesized through which the DNA is transferred. The intercellular transferosome, which is a form of type IV secretion system, is composed of at least eight but often more different proteins (Christie et al., 2014; Christie, 2016; Li et al., 2019; Waksman, 2019). Moreover, most of these proteins are present in multiple copies in the transferosome. The plasmid is recruited to the transferosome through interactions of an ATPase, named coupling protein (CP) and which is located at the cytoplasmic entry side of the transferosome, and the relaxosome proteins. Next, the relaxase pilots transfer of the ssDNA into the recipient cell in an energy consuming process that involves, besides the CP, one or two additional ATPases. Stage 4) is the establishment of the DNA once it has entered the recipient cell. Once transferred, the ssDNA has to be converted into double-stranded DNA (dsDNA). Many conjugative elements encode a primase that is involved in the conversion of ss to dsDNA. Finally, as most bacteria have evolved defense systems that inactivate incoming foreign DNA, including conjugative elements, conjugative elements encode proteins that transiently inactivate these defense mechanisms. So far, little is known about this step and the proteins involved.

Expression of the conjugation genes of most systems are strictly controlled because of at least two reasons. First, expression of the many proteins involved in the conjugation process imposes an energetic burden on the cell. Second, activation of the conjugation process has major consequences for both the plasmid and the host. Conjugative plasmids replicate by the theta type of replication mechanism during vegetative growth of the host. However, replication switches to the rolling-circle mode during conjugation in order to generate the ssDNA molecule that is transferred to the recipient cell (Smillie et al., 2010; Cabezon et al., 2014). Moreover, probably all conjugative plasmids possess a partitioning system to ensure that each daughter cell receives a copy of the plasmid upon cell division (Baxter and Funnell, 2014; Brooks and Hwang, 2017; Bouet and Funnell, 2019). During conjugation, however, the plasmid is recruited to the entry site of the transferosome. The conjugation process also has consequences for the host cell, as it alters characteristics of the membrane and the cell surface.

Studies on conjugative plasmids replicating in G- bacteria have shown that the conjugation genes are strictly controlled by a combination of plasmid and host-encoded factors. In particular, the mechanism controlling conjugation genes of the IncF incompatibility group has been studied in depth (Frost and Koraimann, 2010; Koraimann, 2018). Compared to G− bacteria, conjugation in G+ bacteria is less understood, and little is known about the regulation of conjugation genes (Singh and Meijer, 2014; Kohler et al., 2019). Exceptions are the two related enterococcal plasmids pCF10 and pAD1. Conjugation of these plasmids is induced by pheromones that are produced by plasmid-free recipient cells (for review see, Clewell, 2011; Dunny and Johnson, 2011; Chatterjee et al., 2013; Dunny and Berntsson, 2016).

The 65 kb plasmid pLS20 was originally identified in the *Bacillus subtilis natto* strain IFO3335 (Tanaka and Koshikawa, 1977) and is known to be conjugative in liquid as well as on solid media (Koehler and Thorne, 1987; Meijer et al., 1998; Itaya et al., 2006). Although very little was known about conjugation systems present on *B. subtilis* plasmids, as a host *B. subtilis* is one of the best studied G+ bacteria (Sonenshein et al., 1993; sonenshein et al., 2001). There are also other reasons why *B. subtilis* is an interesting host to study conjugation. *B. subtilis* is closely related to fastidious and pathogenic bacilli, and more distantly related to the Gram+ pathogen *Listeria*. *B. subtilis* is a soil-dwelling bacterium that is found all over the world; therefore pLS20 may be a representative of a group of globally distributed conjugative plasmids. Finally, *B. subtilis* strains are also gut commensals in animals and humans (Cutting, 2011), so it is possible that *B. subtilis* plasmids play a role in HGT in the gut.

Earlier studies on pLS20 have determined the replication region of the plasmid (Meijer et al., 1995), and shown that faithful segregation of the plasmid during cell division is ensured by the actin-like Alp7A protein (Derman et al., 2009). However, little was known about the conjugation process and how it was regulated. In this review we describe the advances that have been made in recent years in understanding how expression of the pLS20 conjugation genes is regulated, and identify the similarities and differences with two other conjugative systems of Gram+ origin. We also discuss future directions to unanswered questions. The most important gaps of knowledge concern structural insights in 1) how repressor molecules generate DNA looping, 2) how the antirepressor interacts with the repressor, and 3) if and how genes involved in different steps of the conjugation process–all located in a single large operon-are differentially expressed. More general reviews of regulation of conjugation in G+ and G− can be found elsewhere (Grohmann et al., 2003; Frost and Koraimann, 2010; Koraimann and Wagner, 2014; Singh and Meijer, 2014; Kohler et al., 2019).

## Evidence That pLS20 Conjugation Is Strictly Controlled and Not Activated by Pheromones

Determination of conjugation efficiencies as a function of growth revealed that maximum conjugation efficiencies, in the range of 1 × 10^−3^, could be obtained only during a short window of time near the end of the exponential growth phase. Conjugation efficiency decreases sharply when donor cells enter the stationary phase, and eventually declines below the detection level (<1 × 10^−8^) (Singh et al., 2013). These results indicate that the conjugation process is strictly regulated. Conjugation efficiencies, which are also low at early time points, increase during the exponential growth phase. However, this increase in efficiency was not due to recipient-produced pheromones though as occurs for the enterococcal pheromone-responsive conjugative plasmids (Dunny, 2013), because similar results were obtained when the growth medium was replaced by fresh medium before mating (Singh et al., 2013). This demonstrated that regulation of pLS20 conjugation is fundamentally different from the enterococcal pheromone-responsive plasmids. The narrow time window of efficient conjugation does not depend on the growth phase of the recipient cells. Thus, pLS20 conjugation is strictly repressed except for a small window of time near the end of the exponential growth phase.

## pLS20 Contains A Large Conjugation Operon That is Preceded By A Divergently Oriented Gene, *rco*_*pLs20*_, Encoding The Master Regulator Of Conjugation

**FIGURE 1:**
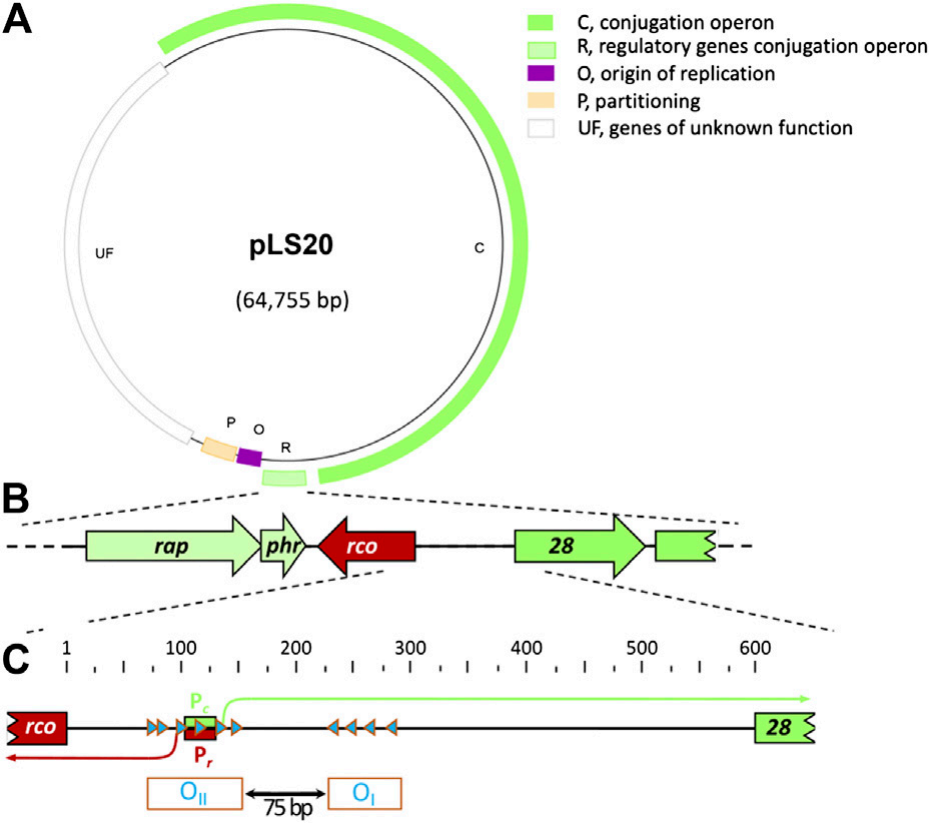
General characteristics of pLS20 and genetic organization of the pLS20 region involved in regulation of the conjugation genes. **(A).** Map of pLS20 (accession number AB615352.1). Different modules are indicated with colors. Light green box, region containing the three genes regulating the activity of the main conjugation promoter P_*c*_ (indicated with “R”); dark green box, conjugation operon (indicated with “C”). Purple box, origin of replication region (indicated with “O”); orange box, partitioning genes (indicated with “P”); and white box, module with unknown genes (indicated with “UF”). **(B).** Blow-up of the region encoding the genes *rap*_*pLS20*_ (*rap*), *phr*_*pLS20*_ (*phr*) and *rco*_*pLS20*_ (*rco*) involved in regulating the expression of the conjugation genes, together with the 5’ region of the conjugation operon. **(C).** Blow-up of the gene *28*-*rco*_*pLS20*_ intergenic region and its features involved in regulating expression of the conjugation genes. The position of the divergently oriented and overlapping promoters P_*c*_ and P_*r*_ are indicated with green and red boxes, respectively. Transcription initiation sites are indicated with bent arrows and the Rco_pLS20_ binding sites with blue triangles. The numbers at the top correspond to bp of the almost 600 bp intergenic region. The operators O_I_ and O_II_, which are separated by a 75 bp spacer, are indicated at the bottom.

Our annotation of the plasmid revealed a large putative operon spanning genes 28-74 in which some genes share similarity with conjugation genes (Singh et al., 2013; see Figure 1A for a schematic view of plasmid pLS20). This indicated that genes 28-74 constitute a large conjugation operon, which was later confirmed (see below). A single divergently oriented gene, 27c, is located immediately upstream of the conjugation operon. Its product, protein p27c, is a putative Xre-type transcriptional regulator predicted to contain a typical DNA binding Helix-Turn-Helix (HTH) domain near its N-terminus. Protein p27c has been shown to be the master regulator of pLS20 conjugation and was named Rco_pLS20_ (regulator of conjugation of pLS20, see Figure 1B) (Singh et al., 2013). Ectopic overexpression of *rco*_*pLS20*_ (= gene *27c*) from a chromosomal location blocked conjugation, and conjugation levels were constitutively high in a derivative of pLS20 lacking the gene (pLS20rco). Plasmid pLS20rco was very unstable in wild type *B. subtilis* cells and its integrity required the ectopic expression of gene *rco*_*pLS20*_ in the host, suggesting that constitutive expression of the conjugation operon is generating high levels of stress to the host. Evidence that Rco_pLS20_ is a transcriptional regulator was obtained by RNAseq analyses (Singh et al., 2013). Thus, genes within the conjugation operon were expressed among the highest level of all plasmid-encoded genes when samples were taken from donor cell cultures at late exponential growth phase, but their levels were >16 fold lower for samples of cells in which *rco*_*pLS20*_ was ectopically overexpressed.

## Promoters Driving Expression of *rco*_*pLS20*_ and the Conjugation Operon

Several analyses, including RNAseq, deletion analyses and primer extension experiments revealed that the promoters driving expression of the divergently oriented genes *rco*_*pLS20*_ and gene *28*, the first gene in the conjugation operon, are located in the 595 bps intergenic region (Ramachandran et al., 2014; see also Figure 1). The promoter upstream of *rco*_*pLS20*_ is named P_*r*_, and the one upstream of the conjugation operon P_*c*_. The P_*c*_ promoter is located 462 bp upstream of gene *28* and, interestingly, overlaps with the divergently oriented P_*r*_ promoter. The strength of the two promoters has been determined using transcriptional *lacZ* fusions (Ramachandran et al., 2014). These analyses showed that the conjugation promoter P_*c*_ is a strong promoter, and promoter P_*r*_, driving expression of *rco*_*pLS20*_, is a very weak promoter whose activity was barely detectable. Surprisingly, analyses of additional strains containing, besides a transcriptional *lacZ* fusion, either pLS20 or a copy of *rco*_*pLS20*_ under the control of an inducible promoter, revealed that Rco_pLS20_ does not just repress the strong promoter P_*c*_, but also regulates its own promoter, P_*r*_. At low expression levels Rco_pLS20_ stimulates the activity of P_*r*_ but at high expression level it represses its own promoter (Ramachandran et al., 2014). It is important to mention that the maximum strength of the P_*r*_ promoter is about 50–75-fold lower than that of the P_*c*_ promoter (Singh et al., 2020). In summary, Rco_pLS20_ represses the strong promoter P_*c*_ and simultaneously regulates the activity of its own promoter P_*r*_ (Ramachandran et al., 2014).

## Regulation of P_*c*_ and P_*r*_ by the Tetrameric rco_pLS20_ Involves DNA Looping

The binding regions of Rco_pLS20_ were initially determined by analyzing the response of P_*r*_ and P_*c*_ promoters to ectopically expressed *rco*_*pLS20*_, using strains containing *lacZ* fused to promoter-containing fragments of different lengths (Ramachandran et al., 2014). This approach revealed that proper regulation required the presence of two DNA regions. These two regions contain 10 octamer boxes whose sequences are (nearly) identical to CAGTGAAA (see Figure 1C for a schematic view). In the first region a cluster of six of these boxes, all in the same orientation, are located near, and overlapping with the P_*r*_ and P_*c*_ promoters. The second region contains four clustered boxes whose orientation is opposite to those present in the first region. The two clusters of boxes are separated by 75 bp. Binding of Rco_pLS20_ to these boxes was demonstrated by DNase I footprinting assays, and also by gel retardation experiments using fragments with/out mutation in the octamers (Ramachandran et al., 2014). The regions containing the four and six octamers are named operator O_I_ and O_II_, respectively (see Figure 1C).

**FIGURE 2:**
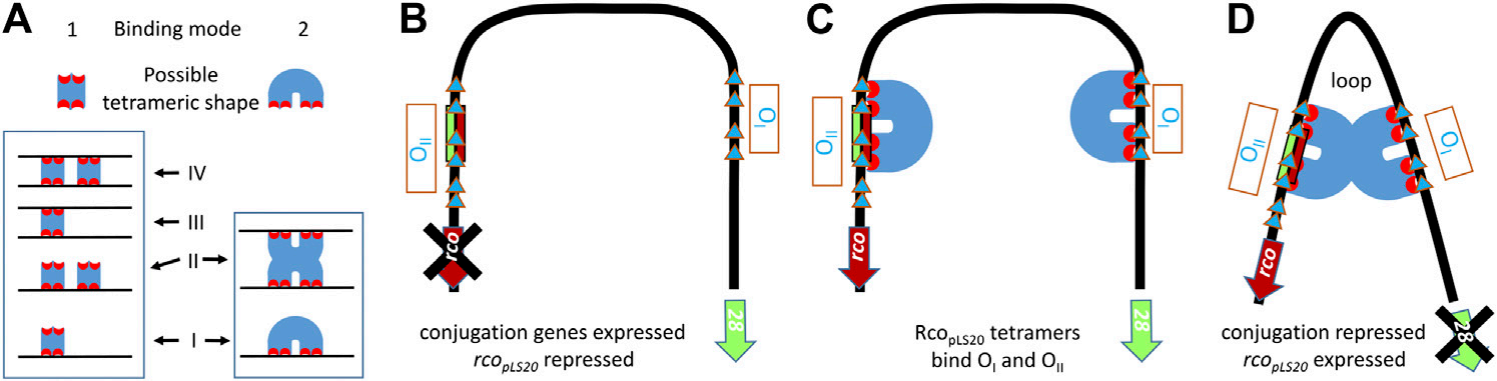
Probable mode of DNA binding by Rco_pLS20_ to the intrinsically bent DNA region encompassing Rco_pLS20_ operators O_I_ and O_II._ **(A).** Schematic representation of two different binding modes by which tetrameric DNA binding proteins can bind a DNA fragment containing one operator. Binding modes 1 and 2 can generate up to four and two different protein-bound DNA configurations, respectively. **(B–D)**. Schematic view of conjugation unrepressed state and repressed state involving DNA looping due to binding of Rco_pLS20_ to operators O_I_ and O_II_ according to binding mode 2 explained in panel **A**. (**B).** Unrepressed state. The conjugation operon starting with gene *28* is derepressed when the operators O_I_ and O_II_ are not bound by Rco_pLS20_ tetramers. **(C).** Rco_pLS20_ tetramers can bind to operators O_I_ and O_II_, resulting in a local high concentration and probably a specific configuration stimulating interaction between the two tetramers. **(D).** Repressed state. Interaction between the two Rco_pLS20_ tetramers bound to operators O_I_ and O_II_ results in repression of the conjugation genes and simultaneous activation of *rco*_*pLS20*_. Blue triangles: Rco_pLS20_ binding sites. Blue U shapes: Rco_pLS20_ tetramers.

The presence of two convergently oriented Rco_pLS20_ operators raised the possibility that binding of Rco_pLS20_ to these operators could result in DNA looping. However, the spacer region between the two operators is only 75 bp. Due to intrinsic stiffness of DNA spacer regions in DNA-loops are generally larger than 90 bp. DNA loops containing smaller spacer lengths are only possible when the DNA region is intrinsically bent. Circular permutation assays showed that the region containing the Rco_pLS20_ operators is indeed intrinsically bent and that the center of the bent is located in approximately the middle of the 75 bp spacer region between operators O_I_ and O_II_ (Ramachandran et al., 2014). Several lines of evidence supported that binding of Rco_pLS20_ to operators O_I_ and O_II_ results in DNA looping. First, ultracentrifugation analysis showed that Rco_pLS20_ forms tetramers in solution (Ramachandran et al., 2014), which is in line with the observation that proteins generating DNA looping commonly form multimers in solution. Second, particularly for short spacer regions, DNA looping requires a certain phasing between the two operators so that operator-bound proteins are in the required configuration to generate the DNA loop. Enlarging the spacer region between the two operators with a half helical turn (i.e., 5 bp) disrupted Rco_pLS20_ mediated regulation of the P_*c*_ promoter *in vivo* (Ramachandran et al., 2014). Finally, Rco_pLS20_-mediated DNA looping was further supported by gel retardation and ultracentrifugation experiments, which also provided important insights into the mode of DNA binding (Singh et al., 2020). DNA looping may be generated as a consequence of one of the two fundamentally different modes by which tetrameric Rco_pLS20_ units bind DNA, as schematically shown in Figure 2A. The difference between these two modes of DNA binding can be distinguished by gel retardation experiments using DNA fragments containing only one operator, as the second binding mode generates a maximum of two retarded species, while the first binding mode would allow the generation of more than two retarded species. Retardations using DNA fragments containing either operator O_I_ or O_II_ both generated a maximum of two retarded species (Ramachandran et al., 2014), indicating that DNA looping occurs through interaction between two Rco_pLS20_ tetramers each bound to an operator. This conclusion was supported by additional gel retardation experiments using two DNA fragments, each containing one operator but having different lengths and distinct flanking sequences, allowing the fragments to be distinguished by Southern blotting. Gel retardation using a mixture of the small and large DNA fragment resulted in the appearance of an additional retarded species composed of a small and a large DNA fragment (Singh et al., 2020). Furthermore, SAXS analysis showed that Rco_pLS20_ forms complexes of increased size at concentrations above 200 μM, indicating that Rco_pLS20_ tetramers can interact with each other (Crespo et al., 2020), which would be expected if DNA binding occurs via the second mode. The combined evidences strongly indicate that efficient repression of the P_*c*_ promoter and simultaneous activation of the P_*r*_ promoter by Rco_pLS20_ is achieved through DNA looping as a consequence of interaction between the Rco_pLS20_ tetramers bound to operators O_I_ and O_II_. We cannot fully excluded the possibility that, in addition to DNA looping, binding of Rco_pLS20_ also introduces other effects on the DNA configuration or orientation.

*rco*_*pLS20*_ is under the control of a very weak promoter (see above). Nonetheless, despite the low levels of Rco_pLS20_ present in the cell, the conjugation process is strictly controlled as the number of transconjugants is below the detection level under conjugation-unfavorable conditions. Probably, this is achieved through DNA looping because it causes a high local concentration of the transcriptional regulator at the right location, which has been shown to be able to increase its specificity and affinity, and concomitantly controls the stochasticity of cellular processes (Vilar and Saiz, 2005; Oehler and Muller-Hill, 2010). Thus, DNA looping allows strict regulation at low protein concentration, while remaining sensitive for accurate activation of conjugation when appropriate conditions –as detailed in the following section-occur. Examples of DNA looping playing a crucial role in transcriptional regulation have been described before. One of the best-studied examples is DNA looping exerted by the CI protein and which is crucial for the genetic switch between the lysogenic and lytic state of the *Escherichia coli* phage λ (for review see, Dodd et al., 2005; Oppenheim et al., 2005). There are clear differences between the DNA looping mechanisms of λ and pLS20. Whereas Rco_pLS20_ forms tetramers in solution, CI forms dimers. In addition, the region separating the operators is about 2.3 kb in phage λ, and 75 bp in pLS20.

## Rco_pls20_-Mediated Repression Is Relieved By The Quorum Sensing-Responsive Anti-Repressor Rap_pls20_ Belonging To The RRNPP Family Of Proteins

Expression of the conjugation genes requires suspension of Rco_pLS20_-mediated repression of the conjugation promoter P_*c*_. In case of pLS20, the repressor Rco_pLS20_ is inactivated by an antirepressor, Rap_pLS20_. Annotation of pLS20cat revealed that a two-gene *rap*-*phr* cassette is located downstream of *rco*_*pLS20*_, with direction of transcription convergent to that of *rco*_*pLS20*_ (see Figure 1B). Rap proteins belong to a large family of signal-peptide receptor proteins of the so-called RRNPP family, which is named after its prototypical members Rap, Rgg, NprR, PlcR and PrgX (for review see, Declerck et al., 2007; Rocha-Estrada et al., 2010; Dunny and Berntsson, 2016; Neiditch et al., 2017). RRNPP proteins, which are encoded by many bacteria belonging to the phylum of Firmicutes, are generally co-transcribed with a *phr* gene encoding the cognate signaling peptide. The Phr signaling peptides are synthesized as a pre-proprotein. After being secreted they are cleaved again to generate the mature peptide, which in most cases correspond to the C-terminal region of the pre-proprotein, and which can be imported into the cell by the oligopeptide permease system (Pottathil and Lazazzera, 2003; Monnet et al., 2016). The chromosome of *B. subtilis* contains eight *rap-phr* cassettes, and some *Bacillus* plasmids also contain such cassettes. Many of these *rap-phr* cassettes, including those on plasmids, function to interfere with developmental processes such as sporulation, competence and degradative enzyme production (Perego, 2001; Bongiorni et al., 2006; Smits et al., 2007; Parashar et al., 2013). Ectopically overexpressing the *rap-phr* cassette of pLS20 in *B. subtilis* did not seem to affect developmental processes of the host. However, it strongly affected the conjugation process, resulting in 1) increased maximum conjugation levels, 2) overexpression of genes located in the conjugation operon, and 3) allowing conjugation to occur during a much broader time window. Moreover, conjugation levels were severely affected for a pLS20 derivative lacking *rap-phr* (Singh et al., 2013). Addition of a synthetic peptide corresponding to the five C-terminal residues of the unprocessed Phr peptide also inhibited conjugation, showing that the activity of Rap is regulated by the mature signaling peptide (Singh et al., 2013). Based on these results the genes were named *rap*_*pLS20*_ and *phr*_*pLS20*_ and the mature Phr peptide was referred to as Phr*_pLS20_.

## Minimal *In Vivo* Regulatory Circuit of pLS20 Conjugation

The results outlined above suggest that Rco_pLS20_, Rap_pLS20_ and Phr*_pLS20_ are the only pLS20-encoded proteins involved in regulation of the conjugation genes. This was confirmed using an *in vivo* system in which the regulatory components were uncoupled from their native setting in a *B. subtilis* strain where the *rco*_*pLS20*_ and *rap*_*pLS20*_ genes are placed under different inducible promoters, and a *lacZ* reporter gene fused to the main conjugation promoter P_*c*_. The main conjugation promoter P_*c*_ was active in the absence of both *rco*_*pLS20*_ and *rap*_*pLS20*_ expression. P_*c*_ was repressed when *rco*_*pLS20*_ alone was induced, strongly supporting the view that Rco_pLS20_ is directly responsible for repressing the P_*c*_ promoter, which is in line with *in vitro* results (see above). The P_*c*_ promoter became active when both *rco*_*pLS20*_ and *rap*_*pLS20*_ were induced, showing that Rap_pLS20_ alone is sufficient to relieve Rco_pLS20_-mediated repression of the P_*c*_ promoter. Finally, when both *rco*_*pLS20*_ and *rap*_*pLS20*_ were induced in the presence of the mature peptide Phr*_pLS20_ (added to the growth medium), the P_*c*_ promoter remained inactive, demonstrating that Phr*_pLS20_ is required and sufficient to inactivate Rap_pLS20_ (Singh et al., 2020).

## Relative Promoter Strengths

**FIGURE 3:**
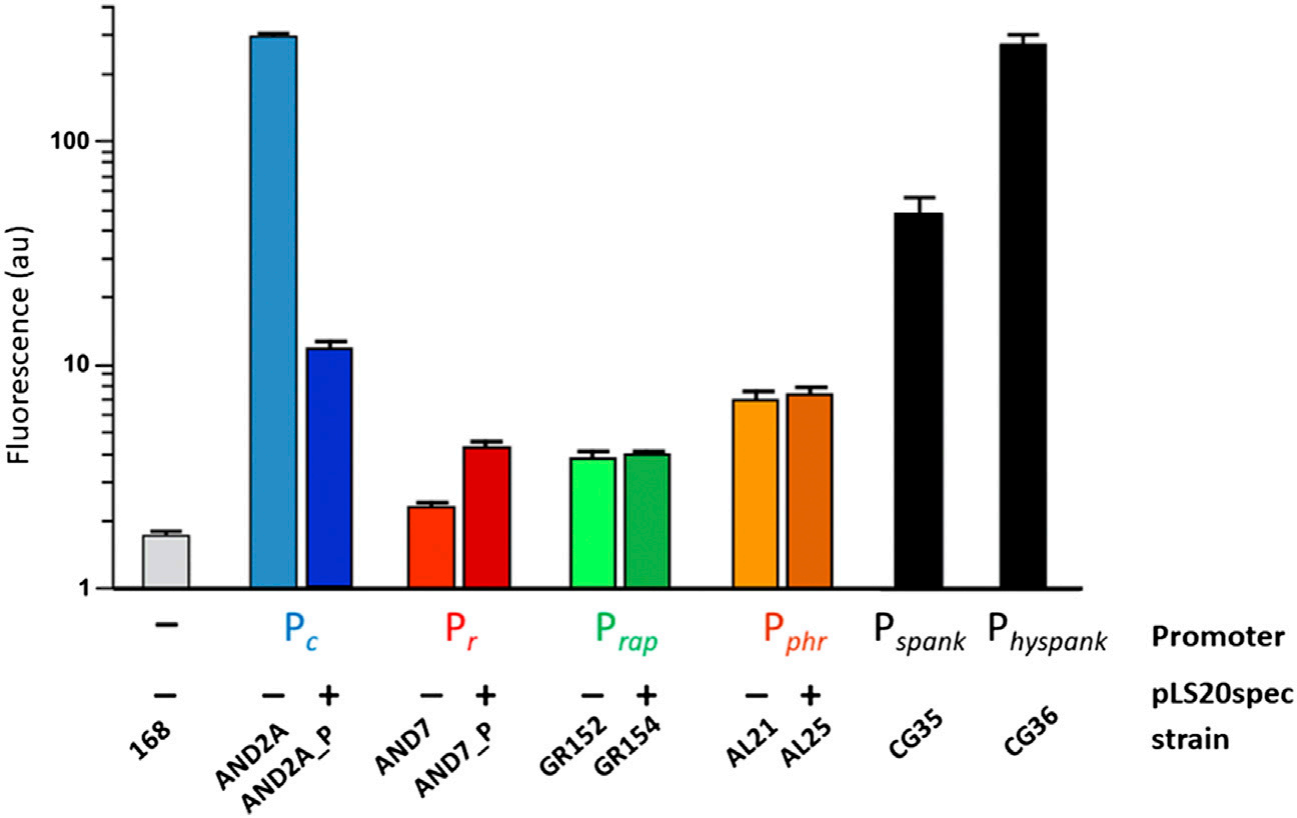
Relative strength of promoters P_*c*_, P_*r*_, P_*rap*_ and P_*phr*_ determined by FACS analysis of strains containing transcriptional *gfp* fusions. Relative promoter strengths determined by cytometry using strains containing promoter P_*c*_, P_*r*_, P_*rap*_ or P_*phr*_ transcriptionally fused to *gfp*. A negative control strain (-) and two positive control strains that contain *gfp* fused to the IPTG-inducible promoter P_*spank*_ or P_*hyspank*_ were included. Values correspond to late exponentially growing cultures (OD_600_ between 0.8-1). Light and dark colored bars reflect strains lacking or containing pLS20spec, respectively. The figure is adapted from (Singh et al., 2020).

The interplay between Rco_pLS20_, Rap_pLS20_ and Phr*_pLS20_ requires an understanding of the relative strengths of their promoters, as well as the activity of promoter P_*c*_, which these proteins regulate. The strengths of P_*c*_, P_*r*_ and the promoter driving expression of *rap*_*pLS20*_ and *phr*_*pLS20*_, named P_*rap*_, were determined using strains containing *gfp* reporter cassettes in which either of these promoters was fused to the *gfp* gene (Singh et al., 2020). The results obtained with the *gfp*-fusion strains confirmed that P_*c*_ and P_*r*_ are a strong and a very weak promoter, respectively. P_*rap*_ was found to be a weak promoter, whose strength was similar to the P_*r*_ promoter in cells containing pLS20, and about 50 to 75-fold lower than that of P_*c*_ (see Figure 3).

P_*rap*_ drives the expression of *rap*_*pLS20*_ and *phr*_*pLS20*_. However, the concentration of Phr_pLS20_ will drop once it is exported from the cell and it is plausible that proper signaling of Phr*_pLS20_ requires higher expression levels than that of Rap_pLS20_. Analysis of an additional strain containing *gfp* fused to the upstream region of *phr*_*pLS20*_ revealed that the expression of *phr*_*pLS20*_ indeed is controlled by a second promoter, named P_*phr*_, whose activity is about two-fold higher than that of P_*rap*_. Unlike the P_*c*_ and P_*r*_ promoters, the activities of P_*rap*_ and P_*phr*_ were not affected by the presence of pLS20 in the cell, indicating that Rco_pLS20_ does not regulate these promoters. The absence of Rco_pLS20_ binding motifs in the vicinity of the P_*rap*_ and P_*phr*_ promoters supports this conclusion (Singh et al., 2020). In summary, 1) the conjugation genes are under the control of the strong P_*c*_ promoter, 2) the *rco*_*pLS20*_ and *rap*_*pLS20*_ genes, driving expression of the proteins regulating the activity of the P_*c*_ promoter, are preceded by very weak promoters, 3) expression of *phr* is controlled by P_*rap*_ and additionally by the two-fold stronger promoter P_*phr*_, presumably to accommodate for the dilution effect upon secretion, and 4) Rco_pLS20_ represses P_*c*_ and regulates the P_*r*_ promoter, but not promoters P_*rap*_ and P_*phr*_.

## Promoter P_*c*_ is Homogeneously Expressed

The multi-layered regulation of the P_*c*_ promoter in combination with the Rap*/*Phr-based quorum sensing mechanism might be expected to cause heterogeneous activation of the P_*c*_ promoter in a population of donor cells. This seemed not to be the case though, because flow cytometry analyses of samples of *B. subtilis* cells having a chromosomal cassette containing the P_*c*_-*gfp* fusion, or those harboring in addition pLS20, both showed homogeneous activity of the P_*c*_ promoter (Singh et al., 2020). In this set up, the P_*c*_ promoter was present on the bacterial chromosome whereas proteins regulating its activity were encoded by the resident plasmid containing its own P_*c*_ promoter. A homogeneous pattern of P_*c*_ promoter activity was also found for cells containing a derivative of pLS20cat in which a copy of the *gfp* gene was placed on pLS20 behind gene *28*, the first gene of the conjugation operon. Together, these results strongly indicate that the multi-levels of regulation results in a sensitive genetic switch that activates the P_*c*_ promoter in a coordinated manner in most or all pLS20-containing cells (Singh et al., 2020). This does not automatically imply that activation of the P_*c*_ promoter guarantees successful plasmid transfer or even the initiation of the conjugation process in all cells. In fact, in most if not all conjugation systems studied, the full conjugation process appears to be activated only in a subpopulation of the donor cells (reviewed in, Koraimann and Wagner, 2014; Stingl and Koraimann, 2017). In the case of pLS20, checkpoints may be present downstream the P_*c*_ promoter. Moreover, successful transfer may not occur even when all conjugation genes are expressed due to, for example, unsuccessful mating pair formation or failure of the plasmid to establish itself in the new host. Moreover, it should be noted that the laboratory conditions under which conjugation is studied are very different from natural conditions in which environmental fluctuations at macro and microscale are likely to affect individual cells or subpopulations.

## Rap_pLS20_ Activates Conjugation By Detaching Rco_pLS20_ From Its Operators and Forming Heterocomplexes With Rco_pLS20_

All members of the RRNPP family of proteins analyzed so far, are all-helical proteins composed of a large C-terminal domain (CTD) and a much smaller N-terminal domain (NTD). Whereas the large CTD interacts with its cognate signaling peptide resulting in conformational changes that affects the activity of the protein, the small NTD constitutes the effector domain that interacts with a target molecule ( D’Andrea and Regan, 2003; Rocha-Estrada et al., 2010; Neiditch et al., 2017). Although the effector domains of all RRNPP proteins are composed of three α-helices, they exert their regulatory functions in three fundamentally different ways. In the first subset of RRNPP proteins these three α-helices have evolved into Helix-Turn-Helix (HTH) DNA binding motifs, which allow them to negatively regulate protein expression by binding to DNA. Examples of such RRNPP proteins are PlcR from *Bacillus thuringiensis* (Declerck et al., 2007) and PrgX from *Enterococcus faecalis* (Shi et al., 2005; Dunny and Berntsson, 2016). Particularly the PrgX protein is interesting in the context of this review because it regulates the conjugation genes present on the plasmid pCF10 (see below). In the second subset of RRNPP proteins, the effector domain has phosphatase activity. In *B. subtilis*, the developmental process of sporulation is induced by the phosphorylated form of the master regulator Spo0A, Spo0A∼P. Spo0A phosphorylation is controlled by a phosphorelay consisting of several kinases and two phosphorelay proteins, Spo0F and Spo0B (Hoch, 1993; Grossman, 1995; Molle et al., 2003; Chastanet and Losick, 2011). Several Rap proteins, including RapA, RapB, RapE, RapH and RapJ, can interrupt the phosphorylation of Spo0A by dephosphorylating Spo0F∼P. RapA was the first Rap member discovered to have this activity and was named Rap, which stands for Response regulator Aspartate Phosphatase (Perego, 2001). Finally, in the third subset of RRNPP proteins, the effector domain binds to its cognate effector protein, which impedes the function of its cognate effector protein (in)directly by modulating the expression of the differentiation pathway involved. Examples of these are RapC, RapF, RapG, RapH and RapK (note that not all Rap proteins have phosphatase activity). It is also worth mentioning that the effector domain of some RRNPP proteins, for instance RapH and NprR, exhibit both activities.

**FIGURE 4:**
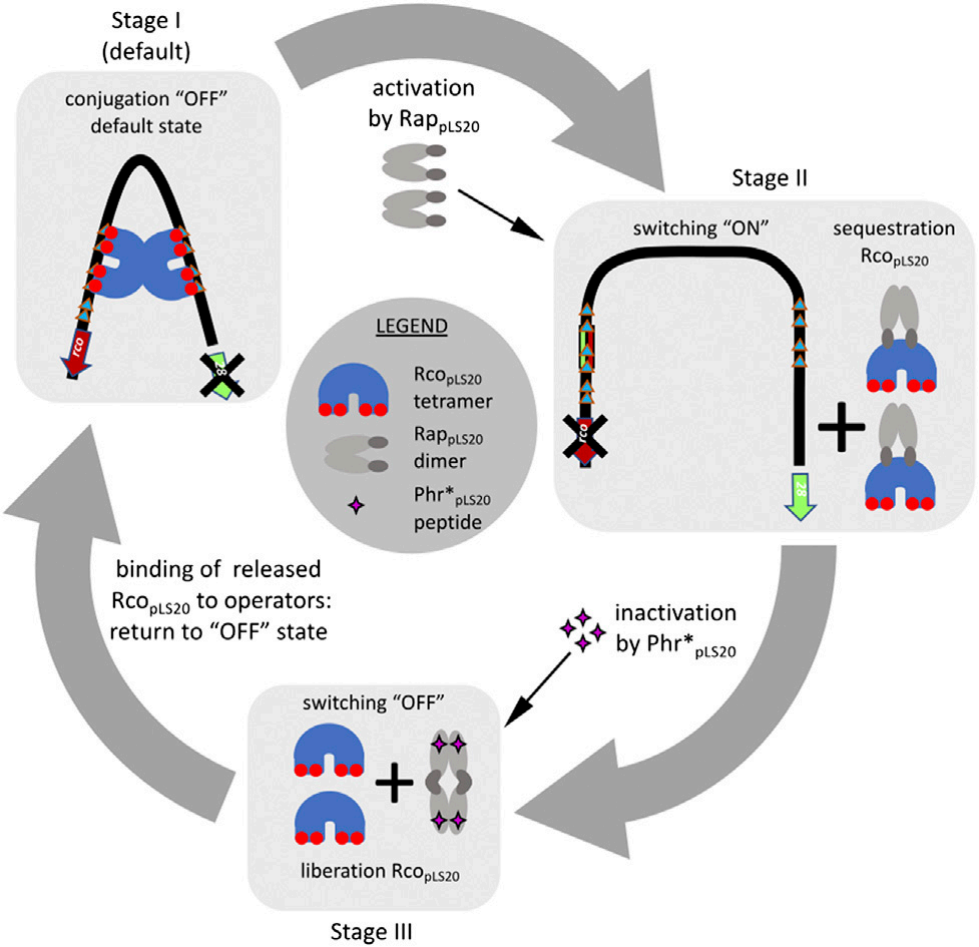
Overview of the current understanding of how the pLS20 conjugation genes are regulated by Rco_pLS20_, Rap_pLS20_ and Phr*_pLS20_. Stage I, as default, the two Rco_pLS20_ tetramers bound to operators _OI_ and O_II_ interact with each other resulting in DNA-looping. This configuration causes a steady state in which the strong conjugation promoter P_*c*_ is repressed and the weak P_*r*_ promoter driving expression of *rco*_*pLS20*_ is simultaneously stimulated. Stage II, the conjugation genes are activated when two apo-form of Rap_pLS20_ dimers detach two Rco_pLS20_ tetramers from the DNA through direct interaction and generating Rap_pLS20_:Rco_pLS20_ heterocomplexes in a stoichiometry of 1:2. Stage III, binding of the Phr*_pLS20_ signaling peptide provokes a conformational change of Rap_pLS20_ that stimulates tetramerization of Rap_pLS20_ via the so-called foot-2-foot configuration in which the N-terminal effector domains interact with each other and are, presumably, no longer available to interact with Rco_pLS20_. The liberated Rco_pLS20_ tetramers are then free to bind its operators thereby returning the system to the default conjugation repressed “OFF” state (stage I).

Based on the features of the known RRNPP proteins, one possibility of how Rap_pLS20_ might activate the pLS20 conjugation genes is by competing with Rco_pLS20_ for binding to DNA near the P_*c*_ promoter. However, both gel retardation and analytical ultracentrifugation (AUC) analyses showed that Rap_pLS20_ has no DNA binding activity, and the NTD of Rap_pLS20_ does not adopt a HTH configuration (Crespo et al., 2020; Singh et al., 2020). Instead, Rap_pLS20_ seems to exert its antirepressor activity by binding directly to Rco_pLS20_, as bacterial two-hybrid analysis showed that Rap_pLS20_ and Rco_pLS20_ interact *in vivo* (Singh et al., 2020). *In vitro* evidence that Rap_pLS20_ interacts directly with Rco_pLS20_ has also been obtained by SAXS and analytical size exclusion chromatography (Crespo et al., 2020). Furthermore, AUC analyses provided conclusive evidence that Rap_pLS20_ forms predominantly dimers in solution. Sedimentation velocity experiments of mixtures of Rap_pLS20_ and Rco_pLS20_ resulted in the appearance of a novel species whose deduced molecular weight was compatible with a complex of one Rap_pLS20_ dimer and one Rco_pLS20_ tetramer (Singh et al., 2020). This conclusion was reinforced by multi-signal sedimentation velocity (MSSV) analysis from which it was determined that the heterocomplex was composed of a stoichiometry of 2.1 mol of Rco_pLS20_ per mol of Rap_pLS20_. More importantly, besides demonstrating that Rap_pLS20_ interacts with free Rco_pLS20_, AUC analyses also showed that Rap_pLS20_ is able to interact with DNA-bound Rco_pLS20_, and that this results in the release of Rco_pLS20_ from the DNA and the concomitant formation of Rap_pLS20_/Rco_pLS20_ complexes. Complementary gel retardation experiments provided evidence that Rap_pLS20_ preferentially interacts with Rco_pLS20_ tetramers involved in DNA looping (Singh et al., 2020). In summary, Rap_pLS20_ appears to activate the conjugation genes by preferentially interacting with Rco_pLS20_ involved in DNA-looping, resulting in the detachment of Rco_pLS20_ from its operators, and forming a heterocomplex in which one Rap_pLS20_ dimer binds one Rco_pLS20_ tetramer. A schematic view of how Rap_pLS20_ activates the conjugation genes is shown in Figure 4.

## The Activation State of The Conjugation Genes is Ultimately Determined By The Signaling Peptide Phr*_pLS20_

The presence of the mature signaling peptide Phr*_pLS20_ suppresses the Rap_pLS20_–mediated activation of the conjugation genes (see above). For other RRNPP members it has been shown that signaling peptides interact directly with the CTDs of their cognate RRNPP proteins, causing inactivation through peptide-induced conformational changes of the RRNPP protein. As outlined below, this is also the case for Rap_pLS20_ and Phr*_pLS20_. Phr*_pLS20_ binds directly to Rap_pLS20_ (see below) and the dissociation constant, *K*_*d*_, for Phr*_pLS20_ was determined to be 2.1 and 7.4 μM by ultracentrifugation and fluorescence anisotropy approaches, respectively (Crespo et al., 2020; Singh et al., 2020). Although AUC analysis showed that Rap_pLS20_ forms predominantly dimers in solution (Crespo et al., 2020, see also above), it seems to have an intrinsic ability to form tetramers at higher concentrations, as 1.1% of Rap_pLS20_ was detected as tetramers in solution at 4.5 μM, and this percentage increased to 3.6% at 25 μM. Interestingly, AUC analysis showed that in the presence of Phr*_pLS20_, Rap_pLS20_ forms almost exclusively tetramers in solution (Crespo et al., 2020). The structures of the apo and peptide-bound forms of Rap_pLS20_ have been determined recently, which show that Rap_pLS20_ forms the typical structure of RRNPP family proteins (Crespo et al., 2020). Rap_pLS20_ is an all α-helical protein (17 antiparallel helices) that is composed of a large 14-helix C-terminal domain (CTD) that can bind the signal peptide, and a much smaller 3-helix N-terminal domain (NTD). The three helices of the NTD do not form a HTH topology, but have an approximate parallel configuration. The N- and CTDs are separated by a 13-residue long flexible loop that connects α-helices three and four. Rap_pLS20_ dimerizes through interactions between the CTDs, and these interactions involve helices H5-H7, H16-H17 and the C-terminal residues.

The structure of the peptide-bound form of Rap_pLS20_ showed that Phr*_pLS20_ binds in a cavity of the CTD. Binding of the Phr*_pLS20_ peptide causes the N-terminal domains to swing outwards thereby favoring Rap_pLS20_ to form tetramers (Crespo et al., 2020). Interestingly, the dimer-dimer interface is principally formed between the NTDs of each dimer, resulting in a so-called foot-2-foot interaction (see stage III in Figure 4 for a schematic view). The small NTDs of RRNPP proteins form the effector domain and this is most likely also the case for Rap_pLS20_, implying that the NTD of Rap_pLS20_ interacts with Rco_pLS20_. Due to the particular foot-2-foot configuration induced upon peptide binding, the N-terminal effector domain is no longer capable of interacting with Rco_pLS20_ to relieve repression, providing the molecular mechanism by which Phr*_pLS20_ inactivates the antirepressor activity of Rap_pLS20_. Phr*_pLS20_ can also bind to Rap_pLS20_ complexed with Rco_pLS20_. This conclusion is based on the results of size exclusion chromatography and SAXS experiments, which showed that the addition of Phr*_pLS20_ peptide to preformed Rap_pLS20_/Rco_pLS20_ complexes resulted in disruption of these complexes and the appearance of elution and SAXS patterns that were similar to those of Rco_pLS20_ alone and Rap_pLS20_-Phr*_pLS20_ complexes (i.e., Rap_pLS20_ tetramers) (Crespo et al., 2020).

## Reciprocal Inhibition of Conjugation and Competence Pathways

Under certain growth conditions, *B. subtilis* can develop the state of natural competence in which cells can bind and adsorb extracellular DNA into the cytosol after which it can be integrated into the bacterial genome via homologous recombination (Dubnau, 1991; Hamoen et al., 2003). The processes of conjugation and competence have in common that they transport DNA via a sophisticated membrane-embedded DNA translocation machinery, but in opposite directions: DNA is exported in the case of conjugation, but imported during competence development. In both cases, only one of the two DNA strands (i.e., an ssDNA strand) is transported, and various other similarities exist between competence and conjugation related ssDNA transfer machines (Chen et al., 2005). However, simultaneous import and export of large ssDNA regions by the same cell may be incompatible for several reasons. For instance, simultaneous production and assembly of the ssDNA translocation machineries for competence and conjugation may interfere with each other and*/*or they may compete for the same cellular positions. In addition, the recombination enzymes synthesized during competence may act on ssDNA of the conjugative element. The following evidence indeed supports that conjugation and competence are incompatible processes and that at least two reciprocal mechanisms have evolved to avoid simultaneous activation of the competence and conjugation pathways in the same cell. Competence development is strongly suppressed in cells containing pLS20 by a plasmid-encoded protein called Rok_pLS20_. Rok_pLS20_ shares similarity with *B. subtilis* Rok, which is a repressor of *comK* that encodes the transcriptional activator of the recombination and the structural competence genes. As for Rok, Rok_pLS20_ suppresses competence by repressing *comK* (Singh et al., 2012).

Like conjugation, competence is also induced by quorum sensing signals. The two-component ComP-ComA system plays a crucial role in the induction of competence: upon sensing the proper signals, the membrane-embedded kinase ComP activates ComA through phosphorylation which then activates competence genes, leading ultimately to the activation of the master regulator gene *comK* (Hamoen et al., 2003). RapF, encoded by the *B. subtilis* chromosome, inhibits stimulation of the competence pathway by interacting with ComA thereby preventing ComA from activating gene expression. *RapF* is cotranscribed with *phrF* that encodes the pre-proprotein which, after being processed, generates the signaling peptide PhrF*. High levels of PhrF* stimulate competence by inactivating RapF. Intriguingly, Phr*_pLS20_ and PhrF* are very similar: residues at positions 1, 3 and 4 are identical; position 2 concerns a conserved substitution of Lysine to Arginine, and position 5 a change from Tyrosine to Isoleucine. PhrF* has been shown to cross talk with Rap_pLS20_ (Singh et al., 2020). Thus, PhrF* can interact with Rap_pLS20_ with a *K*_*d*_ that is only 2.5-fold higher than that of Phr*_pLS20_ as determined by AUC (5.3 and 2.1 μM, respectively), and interaction with PhrF* results in tetramerization of Rap_pLS20_. More importantly, PhrF* is able to inactivate Rap_pLS20_ *in vivo* when tested using the minimal regulatory system of pLS20 conjugation (see above).

## Current View of the Signal-Peptide Mediated Regulation of pLS20 Conjugation

**FIGURE 5:**
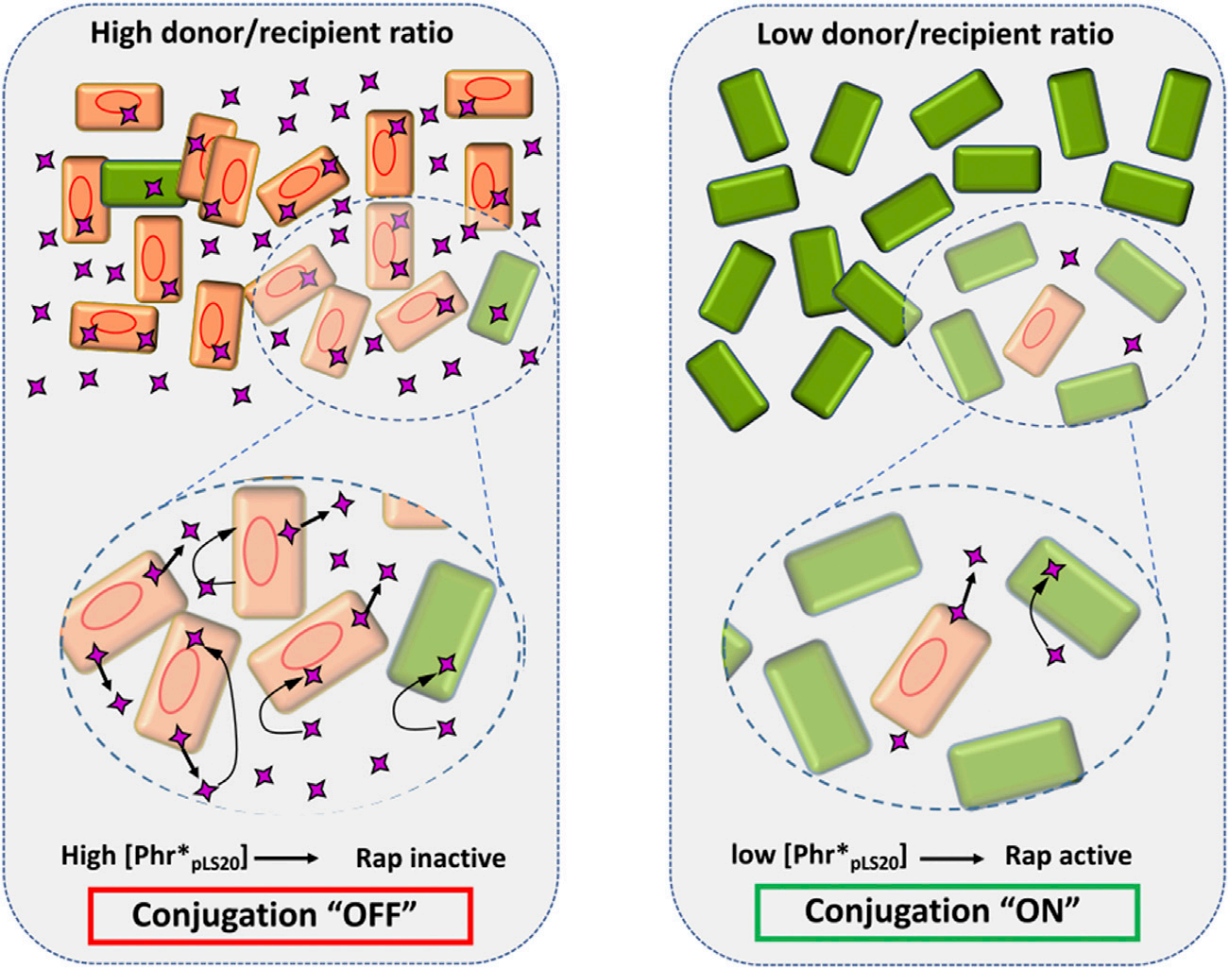
The concentration of the signaling peptide Phr*_pLS20_ determines the activation state of the conjugation genes. Schematic view of how a high and low donor/recipient ratio (left and right panel, respectively) affects the Phr*_pLS20_ concentration, and thereby the activation state of the conjugation process. Donor and recipient cells are indicated with orange and green rectangles, respectively. Red oval lines, pLS20; purple four-pointed star, signaling peptide. The amplified regions in the lower part illustrate secretion of the signaling peptide by donor cells, and its adsorption by donor and recipient cells as indicated by straight and bent arrows, respectively. Note that for simplicity only the mature form of the signaling peptide is shown. See text for details.

All the evidences obtained so far show that the conjugation genes of pLS20 are regulated in a highly sophisticated manner ensuring that conjugation is only activated under conditions that favor a successful conjugation event, i.e. when potential recipient cells are present. A schematic view of the current knowledge of how the conjugation genes are regulated is presented in Figure 4. The main and strong conjugation promoter P_*c*_ located upstream of the conjugation operon is repressed by Rco_pLS20_, which also controls the activity of its own weak promoter P_*r*_ to ensure that only low levels of Rco_pLS20_ are produced. Despite these low levels, the tetrameric Rco_pLS20_ efficiently represses the P_*c*_ promoter. This is most likely achieved through DNA looping that ensures high local concentration of Rco_pLS20_ and simultaneous binding to multiple binding sites in the two operators. The low levels of Rco_pLS20_ probably are also crucial to allow for a sensitive and rapid switch to activate the conjugation genes when favorable conditions occur. Activation of the conjugation genes occurs by the action of the dimeric form of Rap_pLS20_ that relieves Rco_pLS20_-mediated repression of the P_*c*_ promoter. Binding of Rap_pLS20_ to Rco_pLS20_ results in detachment of Rco_pLS20_ from the DNA, and hence it is the apo form of Rap_pLS20_ that activates conjugation. Accumulating levels of Phr*_pLS20_ cause inactivation of Rap_pLS20_ by inducing a conformational change of Rap_pLS20_ that favors the formation of tetramers. Intriguingly, tetramerization occurs through interactions between the N-terminal effector domains with the consequence that these are presumably no longer available to interact with Rco_pLS20_. Consequently, Rap_pLS20_ tetramerization results in the liberation of Rco_pLS20_ from the Rap_pLS20_/Rco_pLS20_ complexes, which are then available to bind its operators and thereby returning the system to the default “OFF” state. Therefore, as schematically shown in Figure 5, the activation state of the pLS20 conjugation genes is ultimately regulated by the concentration of the Phr*_pLS20_ signaling peptide. When all or a majority of the cells within a population contain the plasmid, Phr*_pLS20_ concentrations will be high and conjugation will be inhibited. The conjugation genes will become activated when Phr*_pLS20_ concentrations are low, which occurs when donor cells form a minority of the population and are surrounded by many potential recipient cells. In fact, the presence of recipient cells has a dual effect on lowering the Phr*_pLS20_ concentration: 1) the relative number of donor cells, which produce Phr*_pLS20_, will be lower in populations containing recipient cells, and 2) recipient cells can actively adsorb the signaling peptide. Finally, it is not known if the Rap_pLS20_-Phr*_pLS20_ complexes are destined for rapid degradation, or whether there exists a mechanism to recycle Rap_pLS20_ by removing Phr*_pLS20_ from the complex, thereby stimulating the activation of the conjugation pathway. It should be noted, however, that the promoter P_*rap*_ driving expression of *rap*_*pLS20*_ and *phr*_*pLS20*_ has been shown to be constitutive, thus resulting in *de novo* synthesis of cytosolic Rap_pLS20_, and Phr_pLS20_ that is destined for secretion (Singh et al., 2020).

## Comparison of Regulation of Conjugation Genes of pLS20 and Other Conjugative Elements Regulated By RRNPP Proteins

Signaling peptide receptor proteins belonging to the RRNPP family all have a similar structure that is composed of a small 3-helix N-terminal effector domain and a much larger CTD that, upon interaction with its cognate signal peptide, undergoes a conformational change affecting the functionality of the protein. Despite these conserved features, evolutionary adaptations have led to major effects on the mechanistic action of these proteins. This is exemplified here by comparing the three RRNPP members that regulate the conjugation genes of the conjugative plasmids pLS20 from *B. subtilis*, pCF10 from *Enterococcus faecalis*, and those of the ICE*Bs1* element that is present in many *B. subtilis* strains. The N-terminal effector domain of the pCF10-encoded RRNPP protein PrgX has evolved into a HTH DNA-binding domain. Remarkably, PrgX can interact with two signal peptides that cause opposing effects on its DNA binding. One of these signal peptides, iCF10, is encoded by the plasmid, and the other, cCF10 (the pheromone) by recipient cells. PrgX bound to the plasmid-encoded iCF10 adopts a conformation in which PrgX binds DNA resulting in repression of the conjugation genes. However, when PrgX binds to cCF10, DNA is released resulting in activation of the conjugation genes (for recent review see, Dunny and Berntsson, 2016). The regulatory system of pCF10 differs therefore in two important aspects from that of pLS20. First, in the case of pCF10 recipient cells are directly responsible for activation of the conjugation genes, and second, regulation of the conjugation genes is controlled by one instead of two regulatory proteins (PrgX vs. Rap_pLS20_ and Rco_pLS20_).

The conjugation genes of ICE*Bs*1 are repressed by a regulator named ImmR; an analogue of Rco_pLS20_. Relieve of ImmR-mediated repression can occur in two ways. First, as a consequence of RecA-dependent SOS response to DNA damage, or second, by a mechanism that involves the ICE-encoded RRNPP protein RapI (Auchtung et al., 2007; Bose et al., 2008). However, activation of the conjugation genes by RapI occurs in a different way to Rap_pLS20_. RapI is required but not sufficient to activate the conjugation genes; a protease encoded by gene *immA* that degrades ImmR in a RapI-dependent manner is also needed. The exact mechanism of how RapI stimulates degradation of ImmR by ImmA is not yet known (Bose et al., 2008).

The presence and absence of a protease dedicated to degrade the repressor is likely to have important effects on reversibility of switching on and off of the conjugation pathway. Similar to Rco_pLS20_, ImmR represses the conjugation promoter and simultaneously activates its own promoter (Auchtung et al., 2007). Degradation of ImmR will therefore result in activation of the conjugation genes and concomitantly inhibits *de novo* ImmR synthesis, causing conjugation of the ICE*Bs1* to be an irreversible process. In the case of pLS20, conjugation may be a reversible process, or at least it may be more flexible than the ICE*Bs1* system, because, rather than being degraded, Rco_pLS20_ becomes temporally sequestered by Rap_pLS20_. Inactivation of Rap_pLS20_ by Phr_pLS20_ results in the release of Rco_pLS20_, which can then bind to DNA and again repress the conjugation genes. Interaction of Rap_pLS20_ with Rco_pLS20_ might inactivate Rco_pLS20_ by altering its oligomerization state, in a similar way that RapF causes dissociation of its dimeric interacting partner ComA upon binding (Griffith and Grossman, 2008; Baker and Neiditch, 2011; Wolf et al., 2016). However, results of different analyses compellingly show that Rco_pLS20_ is released as functional tetramers from the Rap_pLS20_-Rco_pLS20_ complex upon addition of Phr*_pLS20_, lending further support for the view that activation of the pLS20 conjugation genes can be a reversible process.

## Unanswered Questions and Future Perspectives

Studies of the past years have resulted in major advances in understanding how the conjugation genes of pLS20 are regulated. Yet, multiple questions remain unanswered. Several of these concern multimerization of Rco_pLS20_ and how Rco_pLS20_ interacts with DNA and with Rap_pLS20_. *In silico* analysis indicates that Rco_pLS20_ contains a HTH motif in its N-terminal region, which probably will be responsible for DNA binding. Therefore, the CTD of Rco_pLS20_ is presumably involved in tetramerization, but how the Rco_pLS20_ monomers interact to form a tetramer is unknown. Currently, it is also not exactly known how two tetramers interact when they are bound to DNA and induce DNA looping. Binding to DNA may generate a conformational change in the tetrameric structure of Rco_pLS20_ that favors octamerization. Alternatively, octamerization may be favored by a local high concentration as the consequence of Rco_pLS20_ binding to the intrinsically bent region containing operators O_I_ and O_II_. The interacting surface between Rco_pLS20_ and Rap_pLS20_ is also not known. Rap_pLS20_ may bind the DNA binding surface of Rco_pLS20_ in an analogous way as RapF interacts with the DNA binding domain of ComA (Baker and Neiditch, 2011). However, the observation that Rap_pLS20_ appears to bind preferentially to Rco_pLS20_ tetramers that are involved in DNA looping (see above) suggests that Rap_pLS20_ binds preferentially, but not exclusively, to the Rco_pLS20_ surface generated upon dimerization of two Rco_pLS20_ tetramers; this interface most likely does not involve the DNA binding domains. Various experimental approaches have shown that Rco_pLS20_ does not bind DNA when it forms a complex with Rap_pLS20_ (Crespo et al., 2020; Singh et al., 2020). This may be because Rap_pLS20_ binds the DNA binding domain of Rco_pLS20_. Alternatively, the formation of an Rco_pLS20_/Rap_pLS20_ complex may induce a conformational change in Rco_pLS20_ affecting its DNA binding ability. Answers to several or all of these questions may be obtained by unraveling the apo structure of Rco_pLS20_ and the structures of Rco_pLS20_ complexed with DNA and with Rap_pLS20_.

The activation of several developmental processes in *B. subtilis* such as sporulation, competence and motility depends on stochastic variability in expression of a master regulator that results in heterogeneic behavior of genetically identical cells within a population (Korobkova et al., 2004; Dubnau and Losick, 2006; Veening et al., 2008). A benefit of heterogeneity can be division of labor, where a subpopulation of cells produces products for the benefit of the entire community. Heterogeneity may also lead to the so-called bet-hedging strategies in which a subpopulation of differentiated cells is generated even in the absence of conditions favoring the differentiation process. Like division of labor, this strategy is beneficial for the community at the population level because the differentiated cells are adapted to adverse conditions that may arise suddenly. However, the process of conjugation is an energy consuming process and has major impacts on cell surface and membrane components, requiring tight repression at times when conditions for successful DNA transfer are not apt. Instead of a heterogeneic behavior resulting in a bet-hedging strategy, the conjugation process must be strictly controlled, allowing its activation to occur only under conditions that are favourable for successful transfer events, and this requires an efficient and sensitive genetic switch from an “OFF” to “ON” state only under conditions that favor successful plasmid transfer. Several features described above contribute to this efficient switch, such as overlapping divergent promoters, low levels of Rco_pLS20_ and Rap_pLS20_, and DNA looping. We consider it possible that the multimerization state of Rco_pLS20_, and particularly the apo and peptide bound forms of Rap_pLS20_ may also contribute to the sensitivity and efficiency of the switch.

Conjugation and competence appear to be incompatible processes. Although no evidence is currently available, one or two-way interference may also exist between conjugative plasmids and phage infection. Some *Bacillus* phages use a quorum sensing mechanism that plays a crucial role in entering the lytic or the lysogenic cycle (Erez et al., 2017). Perhaps, cross-talk exists between this quorum sensing system and the one regulating conjugation.

The work summarized in this review shows that conjugation is exquisitely controlled at the level of the P_*c*_ promoter. However, the conjugation operon is very large and contains many genes that are involved in different stages of the conjugation process, and it is very well possible that subsets of conjugation genes need to be expressed differentially or temporally. This may be achieved, at least in part, by different translation initiation signals, and protein/RNA stability, but we have recently obtained evidence that at least one other layer of expression is involved. pLS20cat gene *29* encodes a surface-located protein that exerts exclusion; i.e., it inhibits futile transfer of the plasmid between donor cells, thereby contributing to efficient transfer to plasmid-free recipient cells (Gago-Cordoba et al., 2019). Proper functioning of exclusion systems require that the gene(s) involved are expressed in all donor cells, but gene *29* is located inside the conjugation operon. This apparent paradox was solved by the identification of a weak promoter located upstream of gene *29*, and a similar strategy is probably used in other conjugative systems as well (Gago-Cordoba et al., 2019). It is possible that the conjugation operon contains additional constitutive or regulated promoters that may modulate the expression of subsets of genes within the operon.

Contrary to most other conjugative systems, pLS20 conjugates efficiently in liquid medium and therefore it will have an important role in gene transfer under natural conditions. However, the predominant lifestyle of bacteria, including *B. subtilis*, is forming colonies or biofilms (Donlan, 2002). pLS20 also conjugates efficiently on solid medium (Koehler and Thorne, 1987; Meijer et al., 1998). As described above, the activation state of the conjugation process is ultimately determined by the concentration of the Phr*_pLS20_ signaling peptide. The results summarized in this review are based on conjugation experiments carried out in liquid medium, and particularly using shaking cultures. Under these conditions, the concentration of the exported signal peptide will be instantly homogenized, which is very different from the situation in which donor cells form part of a colony or biofilm where secretion of the signal peptide will lead to a concentration gradient. It will be interesting to see how the conjugation genes are regulated under such conditions.
